# Assessment of inter-reader agreement and diagnostic performance of Node-RADS in colon cancer

**DOI:** 10.1186/s13244-026-02349-7

**Published:** 2026-07-22

**Authors:** Hui Wang, Bailing Dai, Na Feng, Hongxia Zhang, Xiaoxiang Ning, Kefeng Zhou, Guoqun Mao, Qiang Li, Yongyu An

**Affiliations:** 1 Department of Radiology, Tongde Hospital of Zhejiang Province, Hangzhou, China; 2 Department of Radiology, The First Affiliated Hospital of Zhejiang Chinese Medical University (Zhejiang Provincial Hospital of Chinese Medicine), Hangzhou, China

**Keywords:** Colonic neoplasms, Decision support systems, Lymph nodes, Tomography

## Abstract

**Objectives:**

To assess the inter-reader agreement and diagnostic performance of Node-reporting and data system (Node-RADS) in the evaluation of colon cancer among radiologists with varied experience levels.

**Materials and methods:**

A total of 247 patients with colon cancer were included in this retrospective study. Lymph node evaluations were performed by six radiologists with diverse experience levels (three junior and three senior readers) using Node-RADS. Inter-reader agreement for Node-RADS classification and specific imaging features was assessed using the kappa statistic and compared between junior and senior readers. The diagnostic performance of Node-RADS was evaluated at a cutoff of 3 and 4 score.

**Results:**

Lymph node metastasis was observed in 47.8% of patients. The overall inter-reader agreement was substantial for Node-RADS classification (k = 0.69), almost perfect for size (k = 0.83), moderate for texture and border (k = 0.41–0.45), and fair for shape (k = 0.37). Senior readers demonstrated higher inter-reader agreement in both Node-RADS and specific features than junior readers. Senior readers achieved higher AUC (0.79–0.80) than junior readers (0.72–0.78). At a cutoff of 3, sensitivity, specificity, and accuracy across all readers were 52.5–70.3%, 84.5–93.0%, and 69.2–79.8%, respectively. The corresponding values were 33.9–44.9%, 92.3–98.4%, and 64.4–72.5%, respectively, at a cutoff of 4. Senior readers showed significantly higher pooled sensitivity and accuracy, with comparable specificity compared to junior readers at both Node-RADS cutoffs.

**Conclusion:**

The Node-RADS demonstrates high agreement between observers. Senior readers showed higher inter-reader agreement and diagnostic performance than junior readers. Node-RADS exhibits high specificity but relatively low sensitivity for detecting lymph node metastasis in colon cancer.

**Critical relevance statement:**

This study demonstrated good inter-observer agreement for Node-reporting and data system (Node-RADS) in colon cancer across readers of varying experience, supporting its clinical reliability. However, its limited sensitivity for predicting lymph node metastasis highlights the need for further improvement.

**Key Points:**

- Assessing inter-observer agreement among different readers for Node-RADS assignment is critical before its clinical application.
- Substantial inter-observer agreement for Node-RADS was observed across readers of varying experience levels.

**Graphical Abstract:**

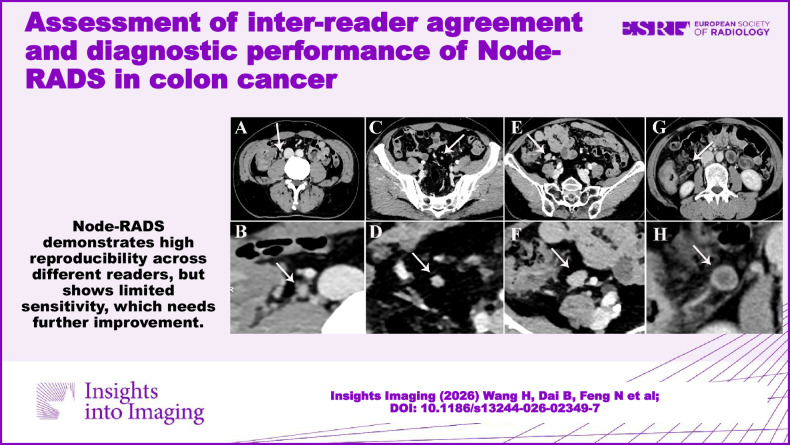

## Introduction

According to data published by the American Cancer Society, approximately 154,270 people will be diagnosed with colorectal cancer and 52,900 will die from this disease in 2025, making it the second leading cause of cancer-related deaths in both men and women [1]. Colon cancer accounts for approximately two-thirds of cases. Lymph node status is a key prognostic determinant in colon cancer, guiding both adjuvant chemotherapy decisions and the evolving use of neoadjuvant therapy [2–4]. The growing evidence for neoadjuvant therapy necessitates precise identification of nodal involvement to optimize patient selection and minimize overtreatment.

Contrast-enhanced computed tomography (CT) remains the most used imaging modality for preoperative staging in colon cancer [5]. However, there is no widely accepted diagnostic imaging criteria for nodal involvement in colon cancer. Diagnostic criteria for nodal metastasis have historically varied, though most rely on short-axis diameter (typically ≥ 10 mm) and morphological features such as irregular borders, heterogeneity, or clustered lymphadenopathy, which have been applied either individually or in combination for diagnosing nodal metastasis in previous studies [6–10]. Nevertheless, CT has limited diagnostic performance for lymph node staging in colon cancer [11], with a pooled sensitivity of 71% and specificity of 67% in a meta-analysis [12]. Therefore, novel approaches are needed to improve the diagnostic performance of preoperative lymph node staging in colon cancer.

With the increasing adoption of reporting and data systems (RADS) in various clinical scenarios, efforts have been made to standardize reports to facilitate communication between clinicians and radiologists, for instance, BI-RADS and PI-RADS. Recently, Node-RADS has been proposed to standardize the radiological assessment of lymph node involvement using predefined criteria and a structured reporting framework [13]. Beyond the size of the lymph node, Node-RADS classification integrates configuration assessment that includes texture, border, and shape [13]. Node-RADS provides a universal approach for lymph node assessment in cancers, independent of tumor origin and nodal anatomy. Several studies have found its value in various cancers, including breast, stomach, kidney, and endometrial cancer [14–17]. A meta-analysis showed the pooled area under the receiver operating characteristic curve (ROC), sensitivity, and specificity of Node-RADS were 0.90, 0.79, and 0.80, respectively [18], indicating that Node-RADS demonstrates great potential in nodal staging in cancers.

Before implementing in clinical practice, Node-RADS should be reliable to minimize variability across readers with different levels of experience. However, considerable heterogeneity was found in previous studies in inter-reader agreement on Node-RADS, with reported kappa values ranging widely from 0.30 to 0.99 [16–20]. Furthermore, most of these studies have only included two raters, which is unreliable for assessing inter-observer agreement. Therefore, this study aims to evaluate inter-reader agreement and diagnostic performance of Node-RADS in colon cancer among radiologists with varied experience.

## Materials and methods

The retrospective study was approved by the Ethics Committee of The First Affiliated Hospital of Zhejiang Chinese Medical University (Zhejiang Provincial Hospital of Chinese Medicine) (No. 2025-KLS-342-01). Informed consent was waived due to the retrospective nature of the study.

### Study cohort

**Fig. 1:**
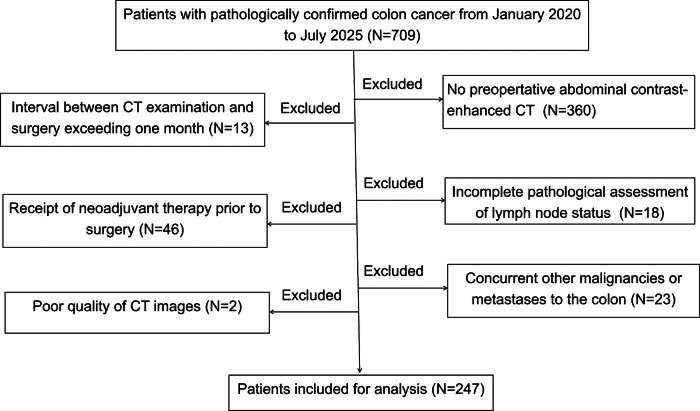
The flowchart of the study cohort

Patients diagnosed with colon cancer at The First Affiliated Hospital of Zhejiang Chinese Medical University (Zhejiang Provincial Hospital of Chinese Medicine) from January 2020 to July 2025 were involved in the study. Inclusion criteria were as follows: (1) patients who underwent abdominal contrast-enhanced CT scan within 1 month prior to surgery; (2) patients who had not received neoadjuvant therapy before surgery; (3) patients with complete postoperative pathological assessment of lymph node status, including the location, number, and unequivocal metastatic status of the harvested lymph nodes. Exclusion criteria were as follows: (1) patients who received neoadjuvant therapy before surgery; (2) patients with incomplete CT images or images of insufficient quality to accurately assess lesions; (3) patients with incomplete pathological assessment of lymph node status; (4) patients with concurrent other malignancies or metastases to the colon. Finally, 247 patients with colon cancer were involved in the study. Figure 1 illustrates the flowchart of the study.

### Image acquisition

Images were performed using multi-slice CT scanners (Siemens Somatom Definition AS, GE Revolution Apex, Philips Spectral CT, and Canon Aquilion ONE). Abdominal imaging protocols followed the manufacturer’s recommendations. All examinations included non-contrast phase, arterial phase, and venous phase acquisitions. For contrast-enhanced scanning, a threshold-triggering technique was employed. The arterial phase was initiated by a 10-s delay after the CT attenuation of the abdominal aorta reached the pre-set threshold of 120 HU, followed by the portal venous phase after a further 25-s delay. Iodinated contrast agent was administered at a rate of 3.0 mL/s with a dose of 1.2 mL/kg body weight. Axial images were reconstructed at 5 mm slice thickness for all phases, with an additional 1 mm thin-section reconstruction for the arterial phase. Coronal and sagittal multiplanar reformations were also generated for image interpretation.

### Image interpretation

**Fig. 2:**
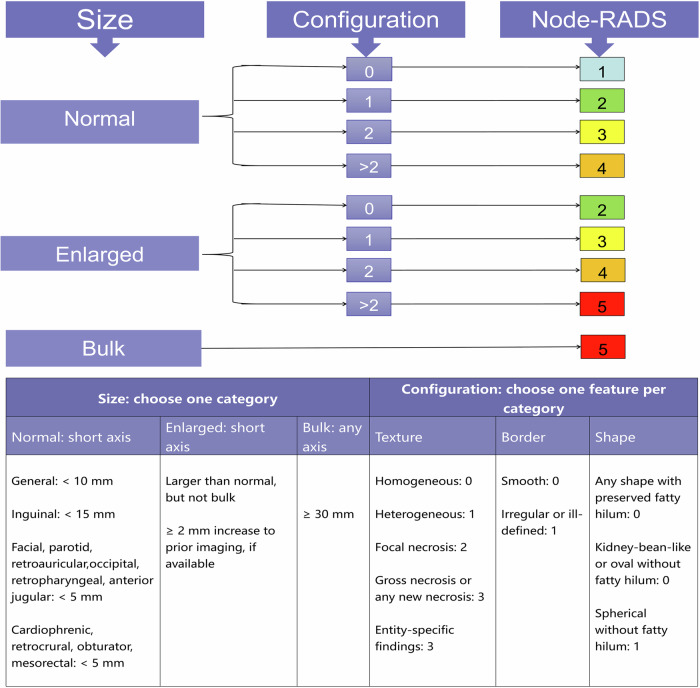
The Node-RADS classification system adapted from the original study

In our study, six radiologists from two different institutions independently evaluated CT images. Among the six readers, R1, R2, and R3, with 1, 3, and 5 years of experience in abdominal imaging, were referred to junior readers. R4, R5, and R6, with 10, 23, and 25 years of experience, were referred to senior readers. They were informed of the tumor location but were blinded to radiological reports and pathological results. Prior to the imaging evaluation, a senior radiologist (R6) provided a concise overview of the Node-RADS based on the original research by Elsholtz et al [13]. This was followed by a collective learning and discussion session between all readers to ensure a thorough understanding of Node-RADS. Additional cases were not provided for practice. Readers were instructed to evaluate and document both lymph node size (normal, enlarged, or bulky) and specific imaging features (texture, border, and shape) for each case. Subsequently, a Node-RADS category was assigned in accordance with the established scoring scheme. In brief, lymph nodes are scored under the guidance of a three-tier flowchart in Node-RADS (Fig. 2). The first two tiers evaluate the two primary imaging criteria: size and configuration. The third tier integrates these findings to assign the final score. Lymph nodes with a short-axis diameter ≥ 10 mm are classified as “enlarged,” while those with any axis ≥ 30 mm are classified as “bulky.” The configuration score is derived from the sum of the ratings assigned to its three subcategories: texture, border, and shape. The final assessment category yields a score ranging from 1 to 5, which reflects the likelihood of malignancy: “1–very low,” “2–low,” “3–indeterminate,” “4–high,” and “5–very high.”

Additionally, prior to the formal image review, a senior radiologist (R6), aware of the tumor location but blinded to the pathological findings, preliminarily assessed CT images to identify and mark the most suspicious lymph node (target lymph node) in each case to ensure consistency in assessing the same lymph node across all readers. During the subsequent independent evaluation, six readers referred to this pre-marked target lymph node for assessment. In case of disagreement over the location of the pre-identified lymph node, a consensus discussion was held among the readers to re-determine the final target lymph node. All evaluations were conducted on a cloud-based imaging platform with anonymized patient identifiers.

### Standard reference

All patients underwent tumor resection combined with systematic lymph node dissection of the locoregional lymph nodes. Cases with pathologically confirmed regional lymph node metastases were classified as node-positive (pN+), and cases without metastasis as node-negative (pN−). Pathological tumor and nodal staging were performed according to the 8th edition of the American Joint Committee on Cancer and the Union for International Cancer Control [21]. In cases where no lymph node involvement was identified, the presence of tumor deposits was considered lymph node-positive (pN+).

### Statistical analysis

All statistical analyses were performed using SPSS Statistics (version 26) and Stata (version 17). Inter-reader agreement for Node-RADS classification was calculated using weighted Fleiss kappa, while agreement for size, texture, border, and shape was evaluated using simple Fleiss kappa. To provide a more comprehensive assessment and avoid the limitations of kappa statistics, both kappa values and the percentage agreement (PA) are presented. The kappa values were interpreted as follows: 0–0.20, slight agreement; 0.21–0.40, fair agreement; 0.41–0.60, moderate agreement; 0.61–0.80, substantial agreement; 0.81–1.00, almost perfect agreement. Inter-reader agreement for Node-RADS and specific imaging features was compared between junior and senior reader groups.

ROC curves were performed to assess the diagnostic performance of Node-RADS classification. The AUCs were calculated for all readers and were compared using the Delong’s test. Sensitivity, specificity, and accuracy of Node-RADS were calculated at both thresholds of 3 and 4 score respectively. Generalized estimating equations were used to compute the pooled sensitivity, specificity, and accuracy for both junior and senior reader groups, and to compare these pooled estimates between groups. A two-sided *p*-value < 0.05 was considered statistically significant for all analyses.

## Results

### Study population

A total of 247 patients were included in the study (mean ages: 66 ± 11 years). All cases were pathologically diagnosed as adenocarcinomas. Pathological lymph node metastases were observed in 47.8% of patients (118/247). There were significant differences in pathological tumor stage between pN− and pN+ (*p* < 0.001). The demographic and clinical characteristics of the patient cohort are summarized in Table 1.

**Table 1:** Demographic characteristics of the included patient population

Characteristics	*N* = 247
Age (mean ± SD, years)	66 ± 11
Gender	
Men	156 (63.2%)
Women	91 (36.8%)
Tumor location	
Cecum	14 (5.7%)
Ascending colon	105 (42.5%)
Transverse colon	21 (8.5%)
Descending colon	27 (10.9%)
Sigmoid colon	80 (32.4%)
pT staging	
T1	17 (6.9%)
T2	37 (15.0%)
T3	121 (49.0%)
T4	72 (29.1%)
pN staging	
N0	129 (52.2%)
N1	82 (33.2%)
N2	36 (14.6%)

### Inter-reader agreement of Node-RADS and specific imaging features

**Fig. 3:**
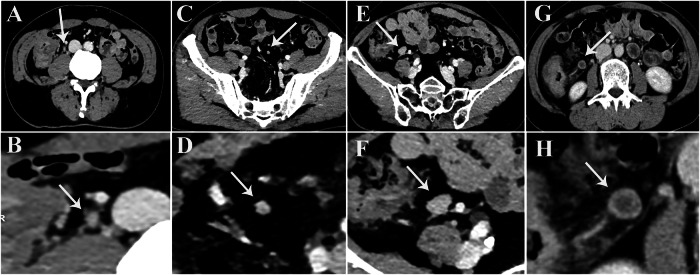
Representative examples of Node-RADS classification. The white arrows indicate the target lymph node. The upper row shows CT images acquired at a standard field of view, while the lower row presents corresponding magnified views of the target lymph node. **A**, **B** Cecal cancer with pT2N0 in a 58-year-old male. All readers evaluated the lymph node as normal size, homogeneous texture, smooth border, and kidney-bean-like shape, corresponding to Node-RADS category 1. **C**, **D** Sigmoid colon cancer with pT4N1 in a 63-year-old male. Evaluation in each reader was as follows: normal size (*n* = 6), heterogeneous texture (*n* = 3), smooth border (*n* = 5), spherical shape (*n* = 3). Node-RADS categories 1–2 were given by four readers, and categories 3–4 were given by two readers. **E**, **F** Ascending colon cancer with pT3N0 in a 73-year old female. Evaluation in each reader was as follows: enlarged (*n* = 6), homogeneous texture (*n* = 6), smooth border (*n* = 4), oval shape (*n* = 5). Node-RADS category 2 was given by four readers, and categories 3–4 were given by two readers. **G**, **H** Ascending colon cancer with pT3N1 in a 59-year-old female. All readers evaluated the lymph node as enlarged, gross necrosis, smooth border, and spherical shape, corresponding to the Node-RADS category 5

Distribution of Node-RADS classification in all readers was as follows: 18.2–48.2% for score 1, 17.8–43.7% for score 2, 12.6–19.8% for score 3, 8.9–13.4% for score 4, and 8.5–10.9% for score 5 (Table 2). The results of inter-reader agreement analysis for Node-RADS classification and specific imaging features are presented in Table 3. For Node-RADS classification, the overall inter-reader agreement was substantial among all readers (PA: 92.6%; k = 0.69, 95% confidence intervals (CI): 0.64–0.73). The overall inter-reader agreement was almost perfect for size (PA: 95.1%; k = 0.83, 95% CI: 0.77–0.89), moderate for texture (PA: 77.4%; k = 0.45, 95% CI: 0.40–0.51) and border (PA: 73.1%; k = 0.41, 95% CI: 0.34–0.48), and fair for shape (PA: 68.4%; k = 0.37, 95% CI: 0.31–0.43). Representative examples are provided in Fig. 3.

**Table 2:** Distribution of Node-RADS classification in all readers

Reader	Node-RADS 1		Node-RADS 2		Node-RADS 3		Node-RADS 4		Node-RADS 5	
	*n*	pN+	*n*	pN+	*n*	pN+	*n*	pN+	*n*	pN+
R1	119	37 (31.1%)	44	17 (38.6%)	34	24 (70.6%)	24	16 (66.7%)	26	24 (92.3%)
R2	88	23 (26.1%)	77	33 (42.9%)	39	21 (53.8%)	22	21 (95.5%)	21	20 (95.2%)
R3	45	10 (22.2%)	111	33 (29.7%)	39	29 (74.4%)	27	21 (77.8%)	25	25 (100%)
R4	82	21 (25.6%)	67	14 (20.9%)	49	36 (73.5%)	25	24 (96.0%)	24	23 (95.8%)
R5	104	25 (24.0%)	59	18 (30.5%)	31	25 (80.6%)	26	25 (96.2%)	27	25 (92.6%)
R6	50	12 (24.0%)	108	31 (28.7%)	33	22 (66.7%)	33	31 (93.9%)	23	22 (95.7%)

Numbers in parentheses are the malignancy rate

**Table 3:** Inter-reader agreement of Node-RADS and specific imaging features

Features	All readers		Junior readers		Senior readers		*p*-values
	PA (%)	Kappa	PA (%)	Kappa	PA (%)	Kappa	
Node-RADS	92.6	0.69 (0.64, 0.73)	91.0	0.62 (0.55, 0.69)	94.4	0.77 (0.72, 0.81)	< 0.001
Size	95.1	0.83 (0.77, 0.89)	93.8	0.78 (0.70, 0.86)	96.8	0.88 (0.82, 0.94)	0.01
Texture	77.4	0.45 (0.40, 0.51)	73.4	0.39 (0.31, 0.48)	79.0	0.46 (0.37, 0.54)	0.259
Border	73.1	0.41 (0.34, 0.48)	70.9	0.37 (0.28, 0.46)	76.8	0.48 (0.39, 0.57)	0.032
Shape	68.4	0.37 (0.31, 0.43)	63.0	0.26 (0.17, 0.34)	75.2	0.49 (0.41, 0.57)	< 0.001

Numbers in parentheses are 95% CIs

*PA* percentage agreement

When stratified by reader experience, the senior reader group showed higher inter-reader agreement than the junior reader group for either Node-RADS or specific imaging features (Table 3). Both groups achieved substantial agreement in Node-RADS (PA: 91.0–94.4%; k = 0.62–0.77). Among the imaging features, size had substantial to almost perfect agreement (PA: 93.8–96.8%; k = 0.78–0.88) in both groups, while the texture, border, and shape showed only fair to moderate agreement (PA: 63.0–79.0%; k = 0.26–0.49).

### Diagnostic performance of Node-RADS classification

**Fig. 4:**
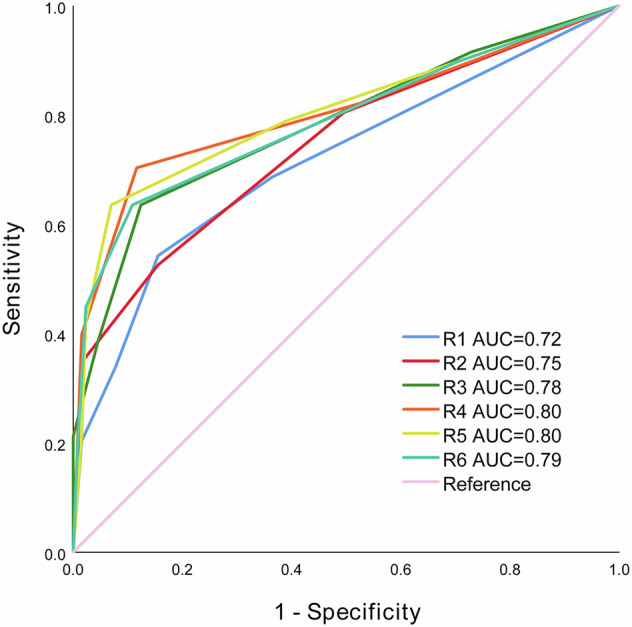
Receiver operating characteristic curve of Node-RADS for each reader. The senior readers showed higher AUCs (0.79–0.80) than those of the juniors (AUCs: 0.72–0.78) when using Node-RADS

The malignant rates across Node-RADS 1–5 in all readers were 22.2–31.1%, 20.9–42.9%, 53.8–80.6%, 66.7–96.2%, and 92.3–100%, respectively (Table 2). The AUCs for the R1-R6 using the Node-RADS were 0.72 (95% CI: 0.65–0.78), 0.75 (95% CI: 0.68−0.81), 0.78 (95% CI: 0.72–0.84), 0.80 (95% CI: 0.74–0.86), 0.80 (95% CI: 0.74–0.86), and 0.79 (95% CI: 0.73–0.85), respectively (Fig. 4). Delong’s test revealed that R1 performed significantly lower than the three senior readers, and R2 was significantly inferior to R4 and R5. However, no statistically significant difference was observed between R3 and the three senior readers.

The diagnostic performance of Node-RADS using cutoffs of 3 and 4 for each reader is presented in Tables 4 and 5. With a Node-RADS score ≥ 3 as the cutoff, sensitivity, specificity, and accuracy in the junior reader group were 52.5–63.6%, 84.5–87.6%, and 69.2–76.1%, respectively, compared with 63.6–70.3%, 88.4–93.0%, and 76.9–79.8% in the senior reader group. The pooled sensitivity and accuracy in the junior reader group were 57% (95% CI: 49–64%) and 72% (95% CI: 67–76%), both significantly lower than those in the senior reader group (66% [95% CI: 58–73%] and 79% [95% CI: 74–83%]; all *p* < 0.001). In contrast, the pooled specificity did not differ significantly between the two groups (86% [95% CI: 80–89%] vs. 90% [95% CI: 85–94%]; *p* = 0.074).

**Table 4:** Diagnostic performance of Node-RADS at a threshold of 3

Reader	TP	FP	TN	FN	Sensitivity	Specificity	Accuracy
R1	64	20	109	54	54.2%	84.5%	70.0%
R2	62	20	109	56	52.5%	84.5%	69.2%
R3	75	16	113	43	63.6%	87.6%	76.1%
R4	83	15	114	35	70.3%	88.4%	79.8%
R5	75	9	120	43	63.6%	93.0%	78.9%
R6	75	14	115	43	63.6%	89.1%	76.9%

*TP* true positive, *FP* false positive, *TN* true negative, *FN* false negative

**Table 5:** Diagnostic performance of Node-RADS at a threshold of 4

Reader	TP	FP	TN	FN	Sensitivity	Specificity	Accuracy
R1	40	10	119	78	33.9%	92.3%	64.4%
R2	41	2	127	77	34.7%	98.4%	68.0%
R3	46	6	123	72	39.0%	95.3%	68.4%
R4	47	2	127	71	39.8%	98.4%	70.4%
R5	50	3	126	68	42.4%	97.7%	71.3%
R6	53	3	126	65	44.9%	97.7%	72.5%

*TP* true positive, *FP* false positive, *TN* true negative, *FN* false negative

When a cutoff score of 4 score (versus 3 score) was used, both junior and senior readers exhibited lower sensitivity and accuracy but higher specificity in their Node-RADS assessments. For the junior readers, sensitivity ranged from 33.9% to 39.0%, specificity from 92.3% to 98.4%, and accuracy from 64.4% to 68.4%. Corresponding values for the senior readers were 39.8–44.9%, 97.7–98.4%, and 70.4–72.5%, respectively. The pooled sensitivity and accuracy of Node-RADS in the junior group was 36% (95% CI: 29–44%) and 67% (95% CI: 61–72%), significantly lower than that in the senior group (42% [95% CI: 35–50%], 71% [95% CI: 66–76%]; all *p* < 0.05), while no statistically differences were found in the pooled specificity between the two groups (95% [95% CI: 92–97%] vs. 98% [95% CI: 94–99%], *p* = 0.086).

## Discussion

The Node-RADS classification, which incorporates both size and configuration criteria, has been introduced in recent years to standardize the assessment of the likelihood of lymph node metastasis in oncology diseases. While multiple studies have validated its diagnostic utility across various malignancies, the consistency and diagnostic performance of Node-RADS among radiologists with differing levels of experience remain underexplored. This study found substantial inter-reader agreement for Node-RADS classification, while agreement on individual imaging features ranged only from fair to moderate. Senior readers showed significantly higher consistency in both Node-RADS classification and individual feature assessment, as well as higher diagnostic performance compared to the junior readers.

While good inter-reader agreement for Node-RADS across different cancer types and imaging modalities (CT/MRI) has been established in the literature, our study specifically confirms that this favorable reproducibility extends to readers with diverse levels of clinical experience. In the study by Li et al [16], two radiologists (with over 7 and 10 years of experience) achieved excellent inter-observer agreement for regional lymph node in papillary renal carcinoma using CT. In another study by Pediconi et al [14], three radiologists (4–15 years of experience) showed substantial or almost perfect agreement with k values of 0.71–0.83 for axillary lymph node in breast cancer using MRI. It should be noted, however, that previous studies exhibited considerable heterogeneity in terms of the number of readers, experience, and use of pre-evaluation training, with most involving only two readers. In contrast, a key strength of our study lies in the involvement of multiple readers with varying levels of experience from two different institutions. Notably, some studies have yielded concerning results on inter-reader agreement. For example, one study in lung cancer reported only moderate agreement (k = 0.48) between readers (with 2 and 3 years of experience) [22], and another in colon cancer found only fair agreement (k = 0.35) [19]. The suboptimal inter-reader agreement observed may be attributable to the limited experience of readers and the absence of training before imaging evaluation in these studies. This postulate is reinforced by the established correlation between higher observer experience and improved agreement, a pattern confirmed in our study and prior studies [20, 23]. Readers without experience in imaging evaluation could achieve good agreement with experienced readers after training [23], underscoring the importance of training for evaluation consistency. Notably, in contrast to previous studies, the most suspicious regional lymph node in our study was initially selected as the target by a senior reader to evaluate the inter-reader agreement of Node-RADS and specific imaging features. The pre-selection of the target lymph node may inflate inter-reader agreement, but this effect should be minimal. In clinical practice, the most suspicious one (usually the largest) is selected by radiologists to assess lymph node status, which is exactly how Node-RADS is intended to be applied. The pre-selection is therefore consistent with both routine practice and the scoring rules. Furthermore, the Node-RADS score dominated by size criteria has a higher predictive value for nodal metastasis than scores dominated by configuration features [24]. This observation aligns well with the clinical behavior pattern. This indirectly suggests that, even if readers were allowed to freely search for the most suspicious node, they would, in the vast majority of cases, select the very same node. Nevertheless, it is not denied that this design may not capture the additional variability introduced by inconsistent lymph node selection in real-world settings. Future studies could adopt either a multi-node or region-based analysis, or a design in which readers independently select the most suspicious lymph node, to better explore the reliability of Node-RADS in clinical practice.

Short-axis diameter, a widely adopted quantitative metric for lymph node evaluation, generally ensures high inter-observer agreement. By contrast, major qualitative imaging features, including texture, border, and shape, demonstrated only fair to moderate inter-reader agreement in our study, consistent with prior studies [15, 19, 23, 25]. Loch et al showed moderate inter-reader agreement on texture and border (k = 0.43–0.46), and only fair for shape (k = 0.23) in gastric cancer [15]. Another study on colon cancer demonstrated similar findings [19]. However, substantial agreement was obtained for these imaging features (k = 0.75–0.77) in a study on esophageal cancer [26]. This variability underscores the inherent subjectivity in assessing qualitative features, which heavily depend on individual interpretive patterns and experience. A contributing factor is that the Node-RADS classification, despite providing example images, lacks operational specifics, such as optimal MRI sequence or CT contrast phase (arterial vs. venous phase), the preferred image thickness (thin-slice vs. thick-slice), and potential interpretation pitfalls. Developing and disseminating more detailed assessment protocols incorporating these details could significantly improve rating consistency.

In the study, AUCs of Node-RADS ranged from 0.72 to 0.80, within the reported values in previous studies. Despite a pooled AUC of 0.90 from a meta-analysis, significant heterogeneity was observed among the included studies [18]. This heterogeneity reflects conflicting results, with the majority demonstrating high diagnostic performance for Node-RADS, while others reported only limited efficacy. For instance, studies reported an AUC of 0.68 in colon cancer [19], 0.60 in papillary thyroid carcinoma [27], and 0.58 in prostate cancer [28]. It is unclear whether tumor type affects the diagnostic performance of Node-RADS, although a recent study on breast cancer showed subtype-dependent differences [29]. Previous studies reported wide ranges of sensitivity (0.22–0.96, pooled values: 0.79) and specificity (0.61–0.96, pooled values: 0.86) [18, 19, 23, 25, 28]. In our study, using a Node-RADS cutoff of ≥ 3, sensitivity and specificity were 52.5–70.3% and 84.5–93.0%, respectively; at a cutoff of ≥ 4, they were 33.9–44.9% and 92.3–98.4%. The wide range of sensitivity and specificity of Node-RADS in studies may be attributable to differences in cancer types, imaging modality, and disease prevalence across study populations. Notably, in our study, Node-RADS showed high specificity but limited sensitivity in colon cancer, with a certain proportion of lymph node metastases being classified as Node-RADS categories 1–2, indicating considerable false-negative results. The findings may result from the intrinsic limitations of Node-RADS, which is fundamentally based on morphology evaluation. When a lymph node harbors microscopic metastatic deposits that have not yet produced recognizable morphological or textural changes, Node-RADS displays negative results. This reinforces the point that Node-RADS in its current form still has room for improvement, and future iterations may benefit from incorporating functional imaging parameters (e.g., apparent diffusion coefficients) to compensate for the limited sensitivity of Node-RADS. That said, these findings do not negate the value of Node-RADS. Like other established reporting systems, such as BI-RADS, Node-RADS provides a standardized reporting framework that facilitates communication among clinicians. Moreover, its diagnostic performance for predicting lymph node metastasis remains superior to that of any individual morphological feature [15, 17, 23, 24, 26]. Additionally, there is no definite threshold to determine lymph node involvement in Node-RADS classification [13], although a threshold of 3 was mostly used in studies. In our study, diagnostic sensitivity and accuracy across all six readers decreased, while specificity increased when the threshold was raised from Node-RADS ≥ 3 to ≥ 4. A threshold of 3 of Node-RADS may be the optimal to assess lymph node involvement in oncological diseases [18, 29].

In the Node-RADS classification, the likelihood of lymph node metastasis increases with a higher score. A score of 1 suggests the lymph node is almost certainly non-metastasis, while a score of 5 indicates it is almost certainly metastasis [13]. However, unlike the BI-RADS classification, Node-RADS does not have predefined malignancy probability ranges. In this study, for each reader, the malignancy rate increased with a higher Node-RADS score. Nonetheless, the malignancy rates for Node-RADS 1 and 2 were 22.2–42.9% across all readers in our study, which is consistent with the limited sensitivity. Previous studies have reported substantial variability in the malignancy rates for Node-RADS categories 1–2, ranging from 0% to 85.7% [15, 16, 19, 22, 29, 30]. A meta-analysis reported a pooled malignancy rate of 4% for category 1 and 31% for category 2 [31]. It is unknown whether factors such as tumor type, imaging modalities, and lymph node location influence the category-wise malignancy rate of Node-RADS. The malignancy rates for categories 3–5 in our study are consistent with previous studies [15, 16, 19, 22, 29–31]. In particular, the malignancy rate for category 5 reached 100%, indicating that lymph node metastasis is almost certain. Regardless, establishing predicted malignancy probability ranges for each category is essential to enhance the clinical utility of the Node-RADS classification. This will standardize radiological reporting and provide clinicians with actionable risk estimates to inform optimal treatment decisions.

There are some limitations in our study. First, the retrospective design inherently introduces potential selection biases, since only patients who underwent radical surgery and pathology-confirmed lymph node status were included. This may limit the generalizability of the findings. Second, all colon cancers included in this study were adenocarcinomas, with no other pathological types represented. Therefore, it remains uncertain whether our findings apply to cancers of different histologies, although this cohort does reflect a common clinical scenario. Furthermore, we did not analyze the diagnostic value of individual imaging features and compare them with Node-RADS, which is beyond the scope of the study. Multiple studies across various tumor types, including gastric cancer and rectal cancer, have already demonstrated that Node-RADS outperforms any individual feature in predicting nodal metastasis [15, 17, 23, 24, 26]. Subsequent studies with larger cohorts focusing on systematic analysis of individual features can be conducted to optimize the scoring structure to improve the performance of Node-RADS. Third, different CT scanners were used in the study; objective image quality metrics, such as signal-to-noise ratio and contrast-to-noise ratio, may indeed vary to some extent across scanners [32], although key image acquisition and reconstruction parameters were kept consistent across all examinations. However, these objective differences do not necessarily result in significant discrepancies in subjective image quality assessment or overall diagnostic capability [33, 34]. More importantly, the key diagnostic features for Node-RADS are predominantly qualitative, with only lymph node size being a quantitative metric. These qualitative morphological assessments are inherently less susceptible to variations in scanner hardware compared to quantitative measurements such as radiomic texture features. Therefore, the impact of scanner type on Node-RADS assessment could be minimal. Multiple CT scanners used in the study also reflect reality in clinical practice. Finally, our study focused on a patient-level binary assessment of regional lymph node status, which is fundamental and clinically important for preoperative surgical planning of lymphadenectomy [35, 36]. We acknowledge that multi-node or region-based analyses could better reflect the value of lymph node staging and provide additional information for postoperative risk stratification and individualized treatment planning. However, such analyses require precise node-by-node correlation between imaging findings and pathological specimens, which was beyond the scope of this retrospective study. Future prospective studies designed with this correlation in mind will enable a more accurate evaluation of Node-RADS at the node or subregion level.

## Conclusion

The Node-RADS demonstrates high inter-observer reproducibility across all experience levels, indicating its robustness in clinical application. Senior readers outperform juniors in both agreement and diagnostic performance. Despite exhibiting high specificity, Node-RADS shows limited sensitivity for predicting lymph node metastasis in colon cancer, and further improvement is needed.

## Data Availability

The datasets used for analyses during this study are available from the corresponding author upon reasonable request.

## Abbreviations

AUC: Area under the curve
CI: Confidence interval
CT: Computed tomography
Node-RADS: Node-reporting and data system
RADS: Reporting and data systems
ROC: Receiver operating curve
